# Supplementary material for: Exploring the relationship between women’s experience of postnatal care and reported staffing measures: An observational study
Source: PLoS One. 2022 Aug 2;17(8):e0266638. doi: 10.1371/journal.pone.0266638 (PMC9345482; doi:10.1371/journal.pone.0266638)
Supplement: S1 File — (DOCX) [file pone.0266638.s001.docx]

## S1. Variable selection to go into the model – testing assumption of independence

The following do not appear to be collinear as the VIF is less than 10, therefore safe to go into model together for each of these outcome measures.

Question : DELAY Question : HELP TIMELY WAY


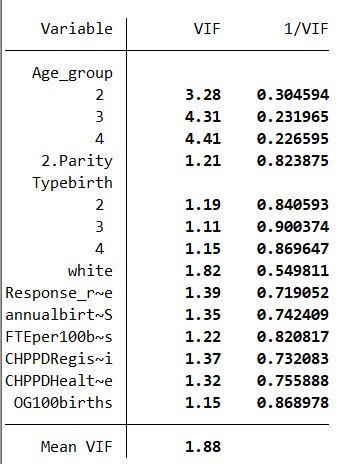

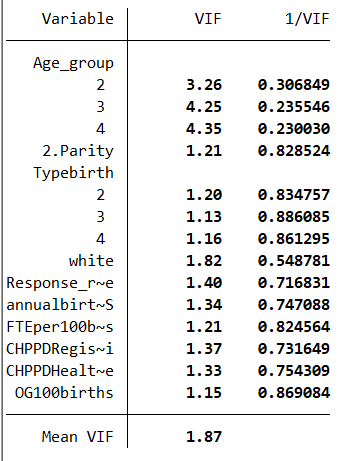


Question : INFO Question : KINDNESS


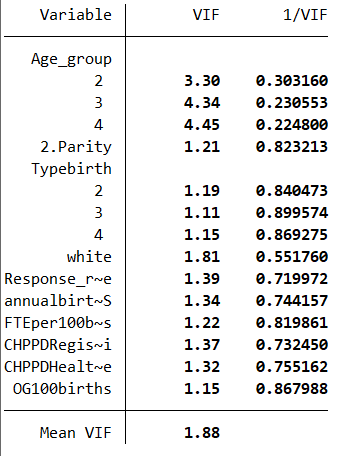

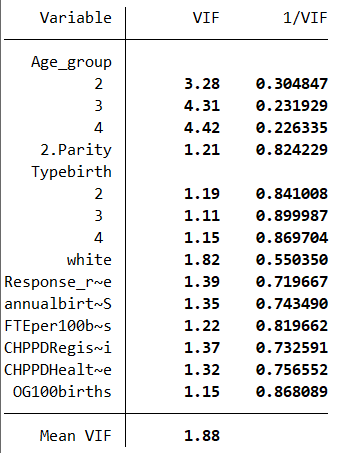


**SELECTING MODELS OF BEST FIT :** Text in red shows model chosen for main analysis

| **NO DELAY** | TRUST MEASURED STAFFING | | |  |  |  |  | AIC | BIC | BIC(93) |  |  |
| --- | --- | --- | --- | --- | --- | --- | --- | --- | --- | --- | --- | --- |
|  | null model | |  |  |  |  |  | 17558.93 | 17573.83 | 17563.98 |  |  |
|  | No_delay_binary ib3.Age_group i.Parity white i.Typebirth FTEper100births | | | | | | | 16856.16 | 16937.82 | 16884.02 | Improved model fit? | |
|  | add response rate | |  |  |  |  |  | 16856.26 | 16945.34 | 16886.66 | No |  |
|  | add number births | |  |  |  |  |  | 16858.15 | 16947.23 | 16888.54 | No |  |
|  | add O&G per 100 births | | |  |  |  |  | 16858.06 | 16947.14 | 16888.45 | No |  |
|  |  |  |  |  |  |  |  |  |  |  |  |  |
|  |  |  |  |  |  |  |  |  |  |  |  |  |
|  | WARD MEASURED STAFFING | | |  |  |  |  | AIC | BIC | BIC(93) |  |  |
|  | No_delay_binary ib3.Age_group i.Parity white i.Typebirth CHPPDRegisteredNursesandMi CHPPDHealthcareSupportWorke \|\|TrustCode:, or | | | | | | | 16989.47 | 17078.65 | 17019.86 | Improved model fit? | |
|  | add response rate | |  |  |  |  |  | 16991 | 17087.61 | 17023.93 | No |  |
|  | add number births | |  |  |  |  |  | 16991.23 | 17087.85 | 17024.16 | No |  |
|  | add O&G per 100 births | | |  |  |  |  | 16859.22 | 16955.72 | 16892.14 | Yes |  |
|  |  |  |  |  |  |  |  |  |  |  |  |  |
|  |  |  |  |  |  |  |  |  |  |  |  |  |
|  | WARD MEASURED : TOTAL STAFF AND SKILL MIX | | | | |  |  | AIC | BIC | BIC(93) |  |  |
|  | No_delay_binary ib3.Age_group i.Parity white i.Typebirth OverallCHPPD PercReg \|\|TrustCode:, or | | | | | | | 16989.11 | 17078.29 | 17019.51 | Improved model fit? | |
|  | add response rate | |  |  |  |  |  | 16990.7 | 17087.31 | 17023.62 | No |  |
|  | add number births | |  |  |  |  |  | 16990.88 | 17087.49 | 17023.8 | No |  |
|  | add O&G per 100 births | | |  |  |  |  | 16858.7 | 16955.2 | 16891.62 | Yes |  |
|  |  |  |  |  |  |  |  |  |  |  |  |  |
|  |  |  |  |  |  |  |  |  |  |  |  |  |

| **HELP** | TRUST MEASURED | |  |  |  |  |  | AIC | BIC | BIC(93) |  |  |
| --- | --- | --- | --- | --- | --- | --- | --- | --- | --- | --- | --- | --- |
|  | null model | |  |  |  |  |  | 15864.99 | 15879.78 | 15870.06 |  |  |
|  | Help_binary ib3.Age_group i.Parity white i.Typebirth FTEper100births | | | | | | | 15210.56 | 15291.48 | 15238.41 | Improved model fit? | |
|  | add response rate | |  |  |  |  |  | 15211.71 | 15300 | 15242.11 | No |  |
|  | add number births | |  |  |  |  |  | 15210.05 | 15298.33 | 15240.44 | No |  |
|  | add O&G per 100 births | | |  |  |  |  | 15212.54 | 15300.82 | 15242.93 | No |  |
|  |  |  |  |  |  |  |  |  |  |  |  |  |
|  |  |  |  |  |  |  |  |  |  |  |  |  |
|  | WARD MEASURED | |  |  |  |  |  | AIC | BIC | BIC(93) |  |  |
|  | Help_binary ib3.Age_group i.Parity white i.Typebirth CHPPDRegisteredNursesandMi CHPPDHealthcareSupportWorke \|\|TrustCode:, or | | | | | | | 15308.74 | 15397.13 | 15339.13 | Improved model fit? | |
|  | add response rate | |  |  |  |  |  | 15310.64 | 15406.39 | 15343.56 | No |  |
|  | add number births | |  |  |  |  |  | 15307.17 | 15402.92 | 15340.1 | No |  |
|  | add O&G per 100 births | | |  |  |  |  | 15210.85 | 15306.49 | 15243.77 | Yes |  |
|  |  |  |  |  |  |  |  |  |  |  |  |  |
|  |  |  |  |  |  |  |  |  |  |  |  |  |
|  | WARD MEASURED : TOTAL STAFF AND SKILL MIX | | | | |  |  | AIC | BIC | BIC(93) |  |  |
|  | Help_binary ib3.Age_group i.Parity white i.Typebirth OverallCHPPD PercReg \|\|TrustCode:, or | | | | | | | 15308.15 | 15396.54 | 15338.54 | Improved model fit? | |
|  | add response rate | |  |  |  |  |  | 15310.07 | 15405.82 | 15343 | No |  |
|  | add number births | |  |  |  |  |  | 15306.55 | 15402.31 | 15339.48 | No |  |
|  | add O&G per 100 births | | |  |  |  |  | 15210.03 | 15305.67 | 15242.96 | Yes |  |
|  |  | | | |  |  |  |  |  |  |  |  |
|  |  |  |  |  |  |  |  |  |  |  |  |  |
|  |  |  |  |  |  |  |  |  |  |  |  |  |

| **INFO** | TRUST MEASURED | |  |  |  |  |  | AIC | BIC | BIC(93) |  |  |
| --- | --- | --- | --- | --- | --- | --- | --- | --- | --- | --- | --- | --- |
|  | null model | |  |  |  |  |  | 16374.35 | 16389.26 | 16379.41 |  |  |
|  | Info_binary ib3.Age_group i.Parity white i.Typebirth FTEper100births | | | | | | | 15474.65 | 15556.26 | 15502.51 | Improved model fit? | |
|  | add response rate | |  |  |  |  |  | 15476.03 | 15565.05 | 15506.42 | No |  |
|  | add number births | |  |  |  |  |  | 15476.39 | 15565.41 | 15506.78 | No |  |
|  | add O&G per 100 births | | |  |  |  |  | 15476.51 | 15565.54 | 15506.91 | No |  |
|  |  |  |  |  |  |  |  |  |  |  |  |  |
|  |  |  |  |  |  |  |  |  |  |  |  |  |
|  | WARD MEASURED | |  |  |  |  |  | AIC | BIC | BIC(93) |  |  |
|  | Info_binary ib3.Age_group i.Parity white i.Typebirth CHPPDRegisteredNursesandMi CHPPDHealthcareSupportWorke \|\|TrustCode:, or | | | | | | | 15594.84 | 15683.96 | 15625.23 | Improved model fit? | |
|  | add response rate | |  |  |  |  |  | 15596.84 | 15693.39 | 15629.76 | No |  |
|  | add number births | |  |  |  |  |  | 15595.89 | 15692.44 | 15628.81 | No |  |
|  | add O&G per 100 births | | |  |  |  |  | 15481.7 | 15578.14 | 15514.62 | Yes |  |
|  |  |  |  |  |  |  |  |  |  |  |  |  |
|  |  |  |  |  |  |  |  |  |  |  |  |  |
|  | WARD MEASURED : TOTAL STAFF AND SKILL MIX | | | | |  |  | AIC | BIC | BIC(93) |  |  |
|  | Info_binary ib3.Age_group i.Parity white i.Typebirth OverallCHPPD PercReg \|\|TrustCode:, or | | | | | | | 15594.88 | 15684.01 | 15625.28 | Improved model fit? | |
|  | add response rate | |  |  |  |  |  | 15596.88 | 15693.44 | 15629.81 | No |  |
|  | add number births | |  |  |  |  |  | 15595.92 | 15692.48 | 15628.85 | No |  |
|  | add O&G per 100 births | | |  |  |  |  | 15481.78 | 15578.22 | 15514.7 | Yes |  |
|  |  |  |  |  |  |  |  |  |  |  |  |  |
|  |  |  |  |  |  |  |  |  |  |  |  |  |

| **KINDNESS** | TRUST MEASURED | |  |  |  |  |  | AIC | BIC | BIC(93) |  |  |
| --- | --- | --- | --- | --- | --- | --- | --- | --- | --- | --- | --- | --- |
|  | null model | |  |  |  |  |  | 14288.45 | 14303.37 | 14293.51 |  |  |
|  | Kind_binary ib3.Age_group i.Parity white i.Typebirth FTEper100births | | | | | | | 13545.28 | 13626.94 | 13573.14 | Improved model fit? | |
|  | add response rate | |  |  |  |  |  | 13546.39 | 13635.47 | 13576.78 | No |  |
|  | add number births | |  |  |  |  |  | 13545.26 | 13634.35 | 13575.66 | No |  |
|  | add O&G per 100 births | | |  |  |  |  | 13547.26 | 13636.35 | 13577.65 | No |  |
|  |  |  |  |  |  |  |  |  |  |  |  |  |
|  |  |  |  |  |  |  |  |  |  |  |  |  |
|  | WARD MEASURED | |  |  |  |  |  | AIC | BIC | BIC(93) |  |  |
|  | Kind_binary ib3.Age_group i.Parity white i.Typebirth CHPPDRegisteredNursesandMi CHPPDHealthcareSupportWorke \|\|TrustCode:, or | | | | | | | 13622.58 | 13711.77 | 13652.98 | Improved model fit? | |
|  | add response rate | |  |  |  |  |  | 13624.31 | 13720.93 | 13657.23 | No |  |
|  | add number births | |  |  |  |  |  | 13621.84 | 13718.46 | 13654.76 | No |  |
|  | add O&G per 100 births | | |  |  |  |  | 13545.12 | 13641.63 | 13578.05 | Yes |  |
|  |  |  |  |  |  |  |  |  |  |  |  |  |
|  |  |  |  |  |  |  |  |  |  |  |  |  |
|  | WARD MEASURED : TOTAL STAFF AND SKILL MIX | | | | |  |  | AIC | BIC | BIC(93) | Improved model fit? | |
|  | Kind_binary ib3.Age_group i.Parity white i.Typebirth OverallCHPPD PercReg \|\|TrustCode:, or | | | | | | | 13622.13 | 13711.32 | 13652.52 |  |  |
|  | add response rate | |  |  |  |  |  | 13623.88 | 13720.5 | 13656.81 | No |  |
|  | add number births | |  |  |  |  |  | 13621.37 | 13717.99 | 13654.3 | No |  |
|  | add O&G per 100 births | | |  |  |  |  | 13544.51 | 13641.02 | 13577.43 | Yes |  |
