# Supplementary material for: Exploring the relationship between women’s experience of postnatal care and reported staffing measures: An observational study
Source: PLoS One. 2022 Aug 2;17(8):e0266638. doi: 10.1371/journal.pone.0266638 (PMC9345482; doi:10.1371/journal.pone.0266638)
Supplement: S3 File — (DOCX) [file pone.0266638.s003.docx]

## S3. Univariable analyses for age group, parity, type of birth, ethnicity, number of births in the Trust, response rate and medical staff

**Question related to being Discharged without delay**

|  |  | Indication of model fit |
| --- | --- | --- |
|  | Empty model | AIC 17558.92 |
| Age group (comparison 30-34) 16- 25 year olds  25 - 29 year olds  35+ year olds | 1.26 (1.09, 1.46) 1.07 (0.97, 1,17) 0.97 (0.89, 1.05) | AIC 17551 |
| Parity (comparison primiparous) Multiparous | 1.43 (1.33, 1.53) | AIC 17228.36 |
| Type birth  (comparison spontaneous birth)  instrumental birth planned caesarean emergency caesarean | 0.67 (0.61, 0.74) 1.08 (0.97, 1.20) 0.87 (0.78, 0.96) | AIC 17259.94 |
| % white ethnicity | 1.00 (1.00, 1.00) | AIC 17559.11 |
| Number of births in Trust per year | 1.00 (1.00, 1.00) | AIC 17559.5 |
| % response rate per Trust | 1.00 (1.00, 1.01) | AIC 17559.71 |
| FTE O&Gper100births | 1.00 (0.77, 1.31) | AIC 17424.37 |

**Question related to Always having help when needed it**

|  |  | Indication of model fit |
| --- | --- | --- |
|  | Empty model | AIC 15864.99 |
| Age group (comparison 30-34) 16 – 25 year olds  25 - 29 year olds  35+ year olds | 0.95 (0.82, 1.11) 1.02 (0.92, 1.13) 1.00 (1.52, 1.80) | AIC 15870.32 |
| Parity (comparison primiparous) Multiparous | .701 (.653, .753) | AIC 15588.74 |
| Type birth  (comparison spontaneous birth)  instrumental birth planned caesarean emergency caesarean | .649 (.583, .723) .691 (.619, .771) .680 (.613, .754) | AIC 15536.37 |
| % white ethnicity | 1.006 (1.001, 1.010) | AIC 15862.09 |
| Number of births in Trust per year | 1.000 (1.000, 1.000) | AIC 15859.81 |
| % response rate per Trust | 1.007 (.997, 1.017) | AIC 15865.23 |
| FTE O&Gper100births | .950 (.670, 1.347) | AIC 15766.32 |

**Question related to Always having Info and explanations**

|  |  | Indication of model fit |
| --- | --- | --- |
|  | Empty model | AIC 16374.35 |
| Age group (comparison 30-34) 16 – 25 year olds  25 - 29 year olds  35+ year olds | 1.01 (0.86, 1.18) 0.97 (0.88, 1.08) 1.09 (1.00, 1.18) | AIC 16374.9 |
| Parity (comparison primiparous) Multiparous | 1.823 (1.691, 1.967) | AIC 15890.25 |
| Type birth  (comparison spontaneous birth)  instrumental birth planned caesarean emergency caesarean | .527 (.475, .587) .738 (.660, .825) .548 (.495, .607) | AIC 15954.53 |
| % white ethnicity | 1.004 (1.000, 1.008) | AIC 16372.26 |
| Number of births in Trust per year | 1.000 (1.000, 1.000) | AIC 16372.45 |
| % response rate per Trust | 1.004 (.995, 1.012) | AIC 16375.57 |
| FTE O&Gper100births | .947 (.706, 1.271) | AIC 16267.14 |

**Question related to Always being treated kindness and understanding**

|  |  | Indication of model fit |
| --- | --- | --- |
|  | Empty model | AIC 14288.45 |
| Age group (comparison 30-34)  16 - 25 year olds  25 - 29 year olds  35+ year olds | 0.76 (0.64, 0.89) 1.03 (0.92, 1.16) 1.04 (0.95, 1.15) | AIC 14279.93 |
| Parity (comparison primiparous) Multiparous | 1.731 (1.592, 1.881) | AIC 13916.3 |
| Type birth  (comparison spontaneous birth)  instrumental birth planned caesarean emergency caesarean | .561 (.500, .631) .627 (.557, .707) .512 (.458, .571) | AIC 13920.35 |
| % white ethnicity | 1.006 (1.002, 1.011) | AIC 14282.91 |
| Number of births in Trust per year | 1.000 (1.000, 1.000) | AIC 14283.7 |
| % response rate per Trust | 1.008 (.999, 1.018) | AIC 14287.22 |
| FTE O&Gper100births | .893 (.639, 1.247) | AIC 14210.48 |
