# Supplementary material for: Exploring the relationship between women’s experience of postnatal care and reported staffing measures: An observational study
Source: PLoS One. 2022 Aug 2;17(8):e0266638. doi: 10.1371/journal.pone.0266638 (PMC9345482; doi:10.1371/journal.pone.0266638)

## S4. Relationship between staffing in Trusts and patient experience (univariable)

Staffing as a continuous variable
**Question related to being Discharged without delay**


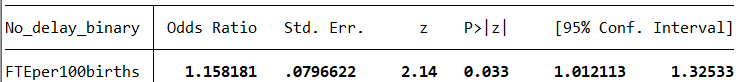


**Question related to Always having help when needed it**
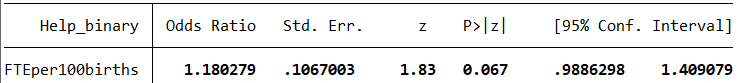


**Question related to Always having Info and explanations**


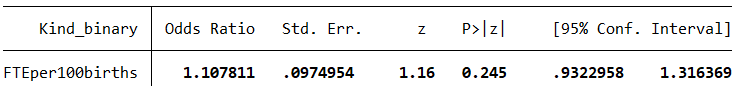
 **Question related to Always being treated kindness and understanding**


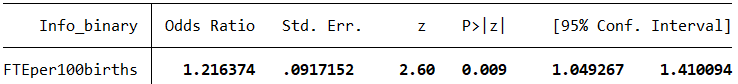


## Mid and Highest tertile compared with lowest tertile as the reference group

**Question related to being Discharged without delay**


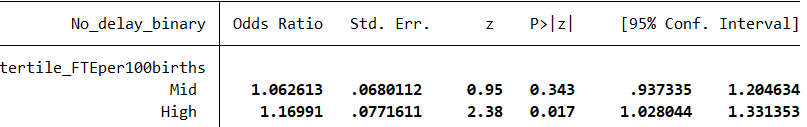


**Question related to Always having help when needed it**


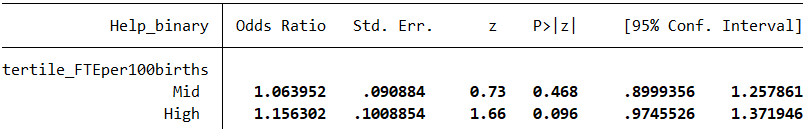


**Question related to Always having Info and explanations**


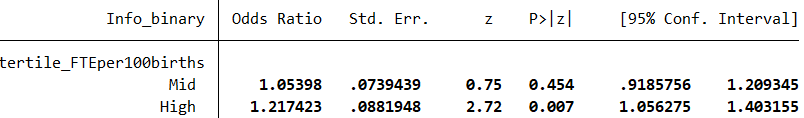


**Question related to Always being treated kindness and understanding**


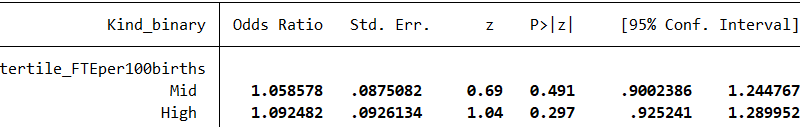

Supplement: S4 File — (DOCX) [file pone.0266638.s004.docx]
