# Supplementary material for: Exploring the relationship between women’s experience of postnatal care and reported staffing measures: An observational study
Source: PLoS One. 2022 Aug 2;17(8):e0266638. doi: 10.1371/journal.pone.0266638 (PMC9345482; doi:10.1371/journal.pone.0266638)

## S7. Relationship between staffing on postnatal wards and patient experience (adjusted models)

**Question related to being Discharged without delay (Individual staff groups)**


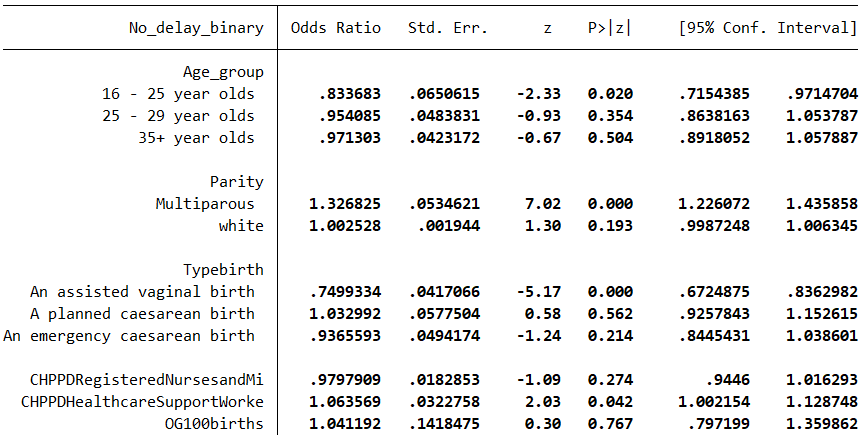


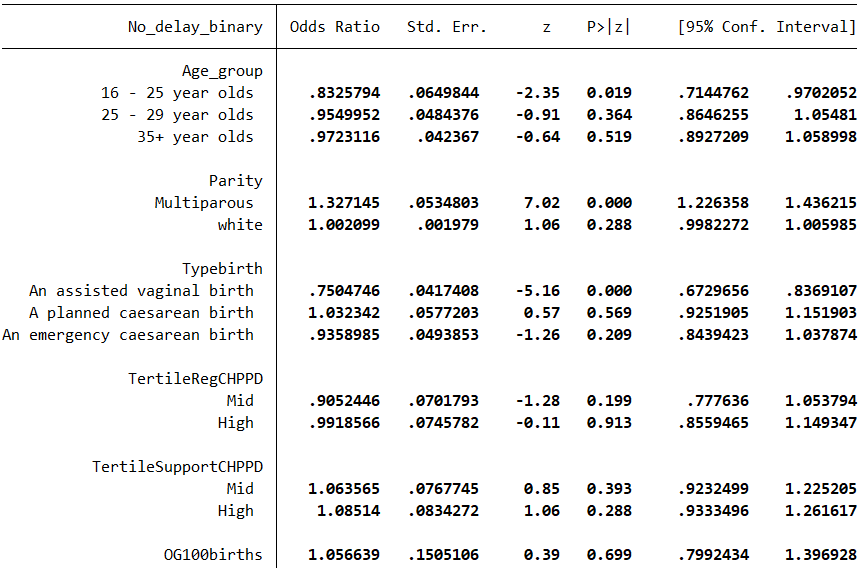


**Question related to Always having help when needed it (Individual staff groups)**


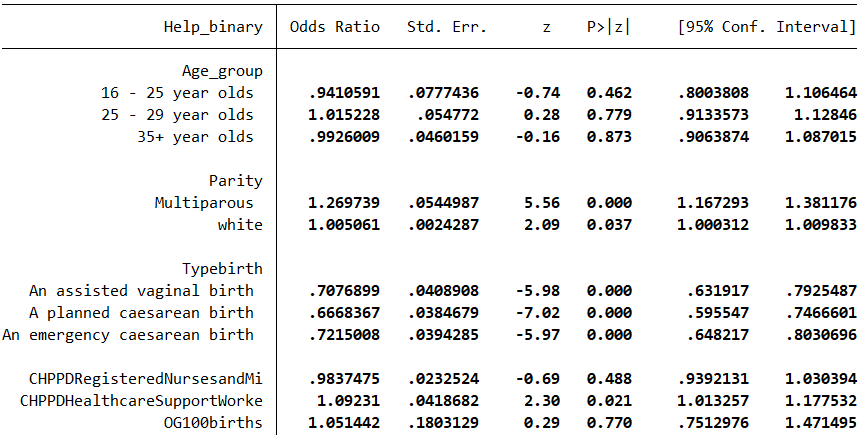


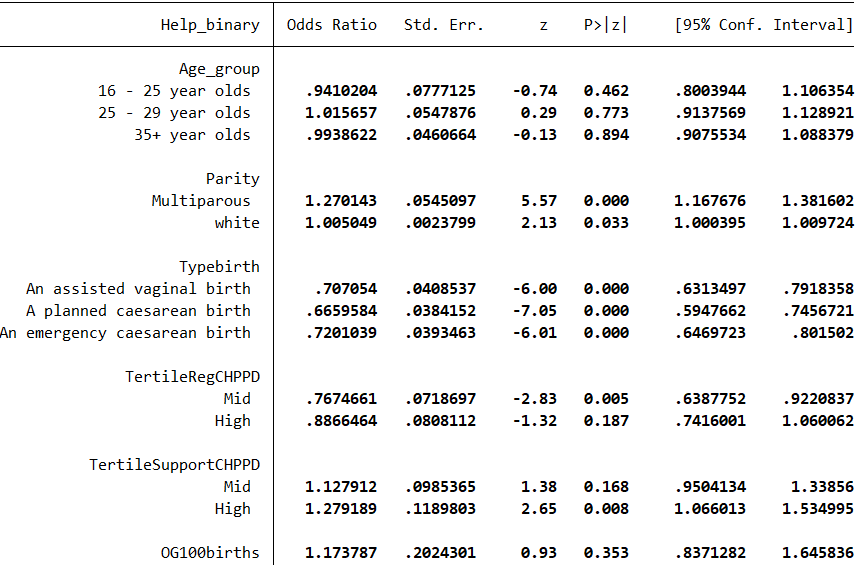


**Question related to Always having help when needed it (Individual staff groups)**


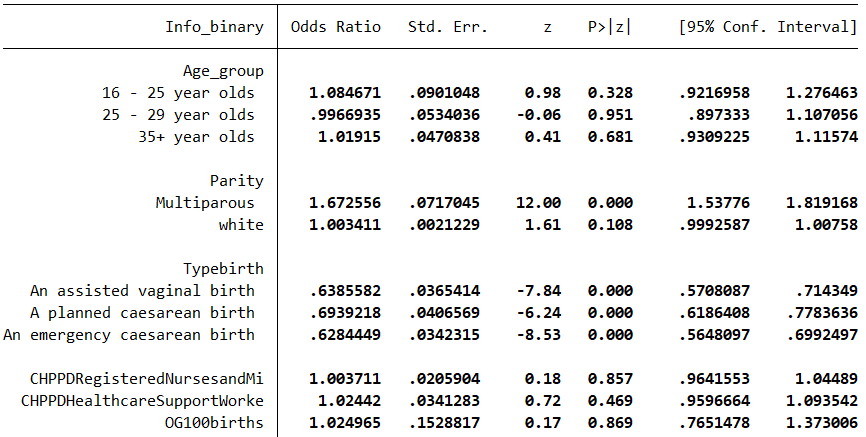


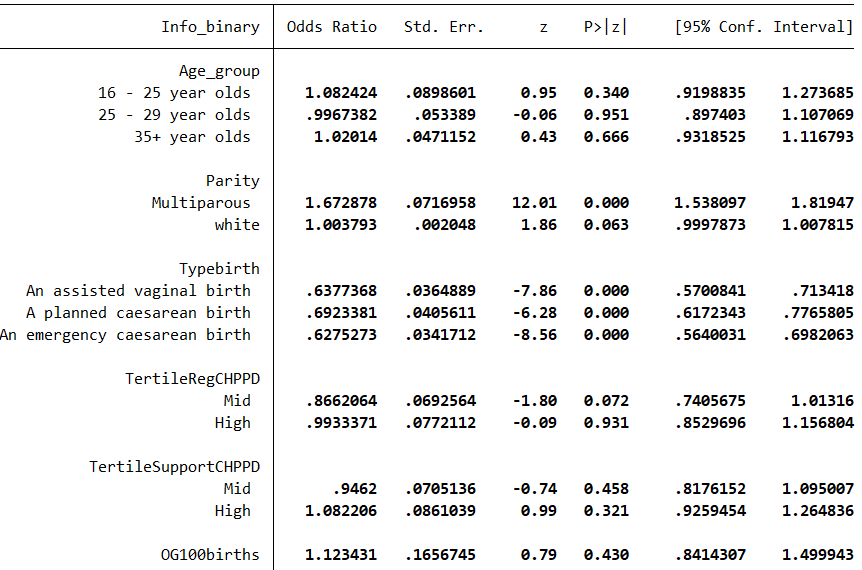


**Question related to Always being treated kindness and understanding (Individual staff groups)**


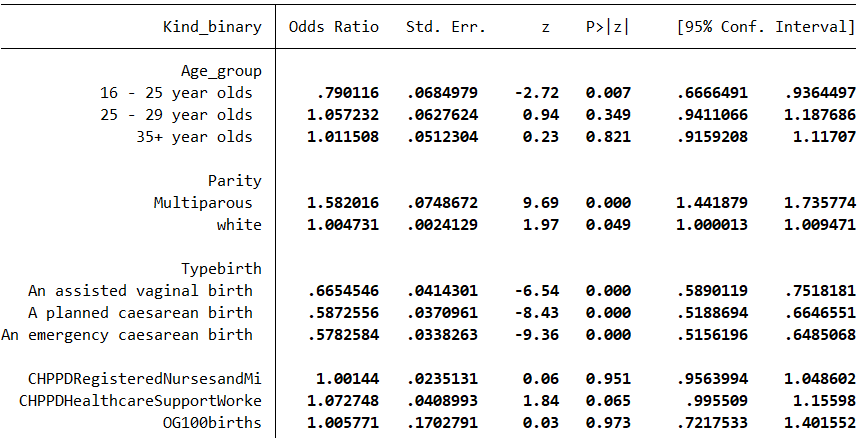


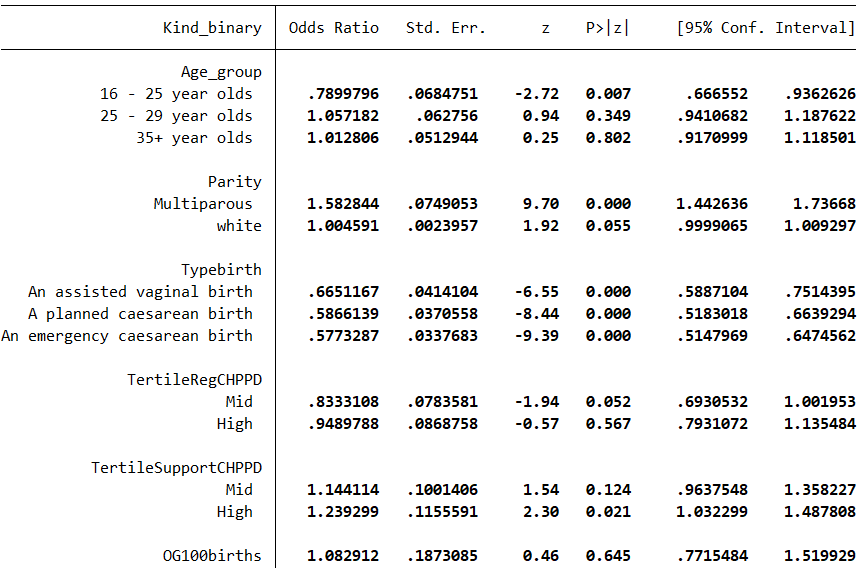

Supplement: S7 File — (DOCX) [file pone.0266638.s007.docx]
