# Supplementary material for: Exploring the relationship between women’s experience of postnatal care and reported staffing measures: An observational study
Source: PLoS One. 2022 Aug 2;17(8):e0266638. doi: 10.1371/journal.pone.0266638 (PMC9345482; doi:10.1371/journal.pone.0266638)

## S8. Relationship between Overall staffing, Skill mix on Postnatal wards and patient experience (adjusted models)

**Question related to being Discharged without delay**
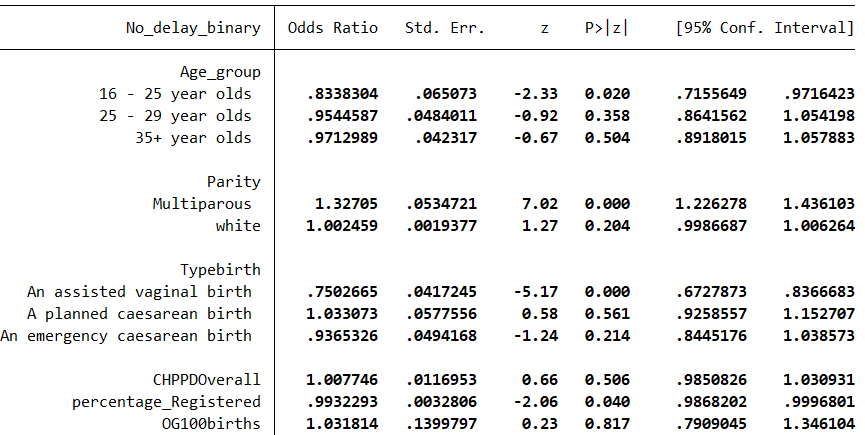


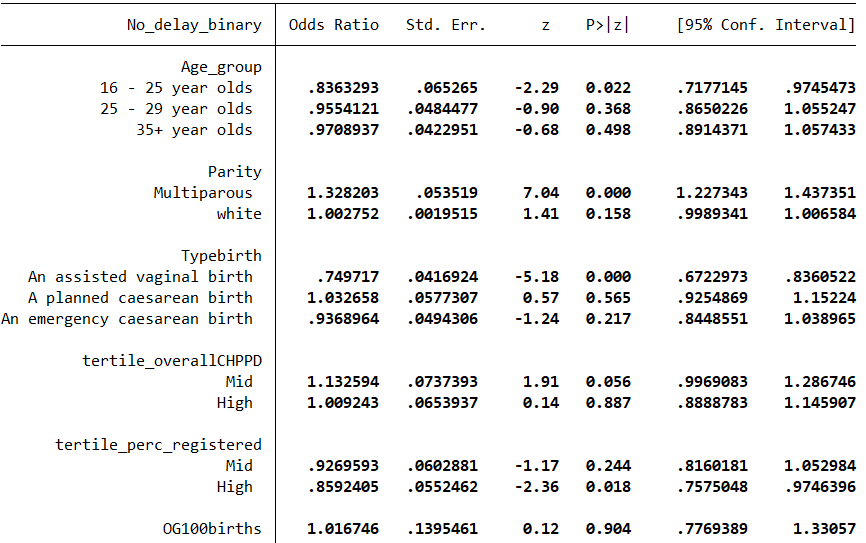


**Question related to Always having help when needed it**


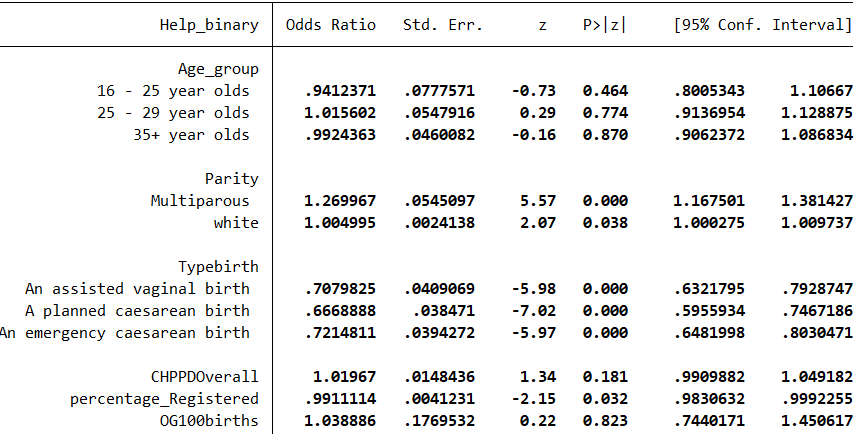


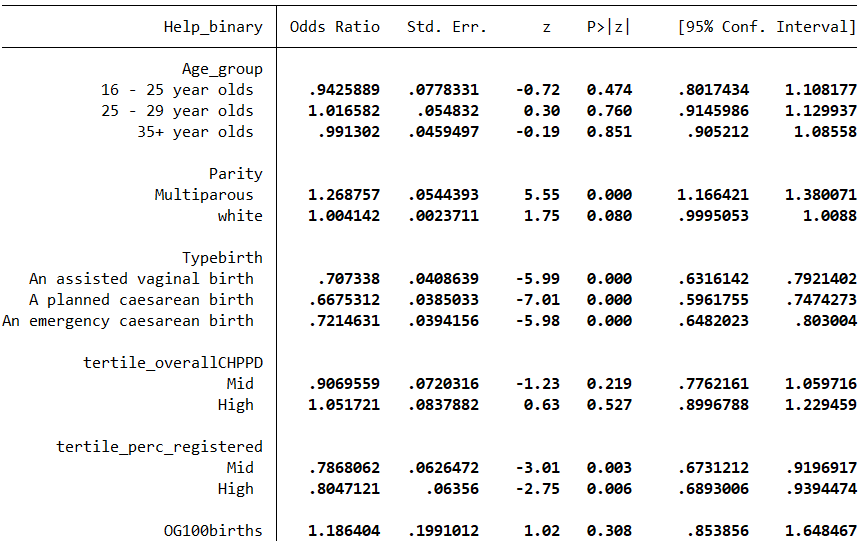


**Question related to Always having Info and explanations**


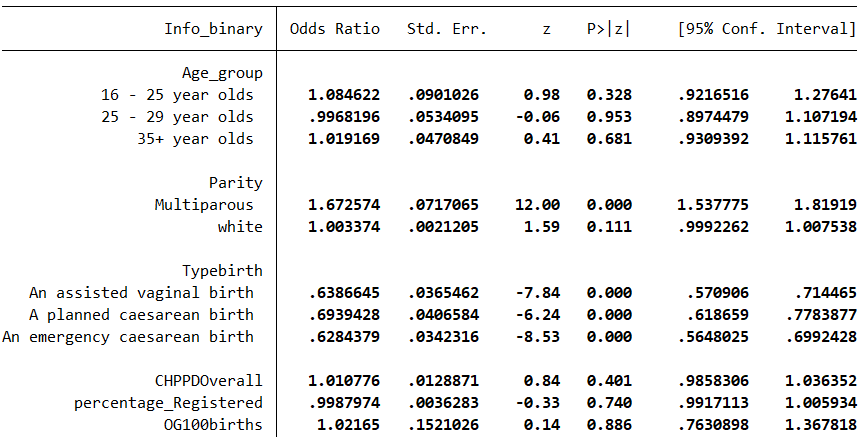


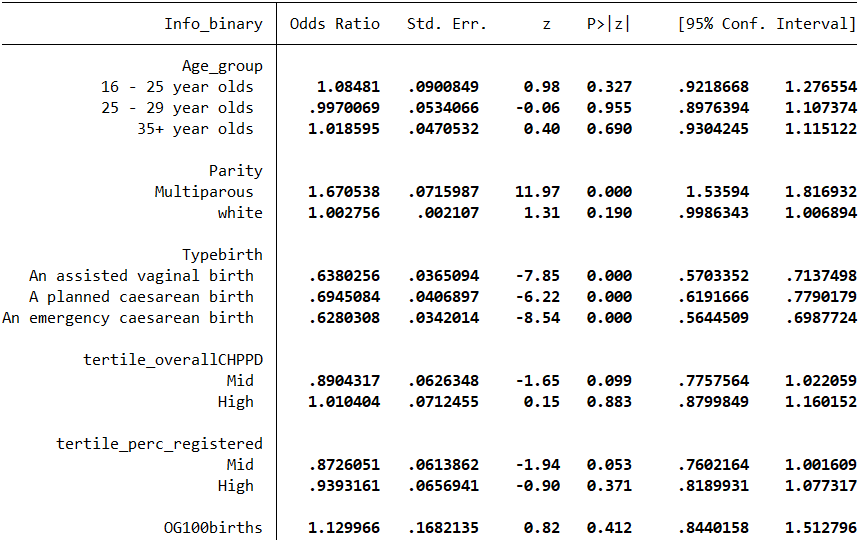


**Question related to Always being treated kindness and understanding**


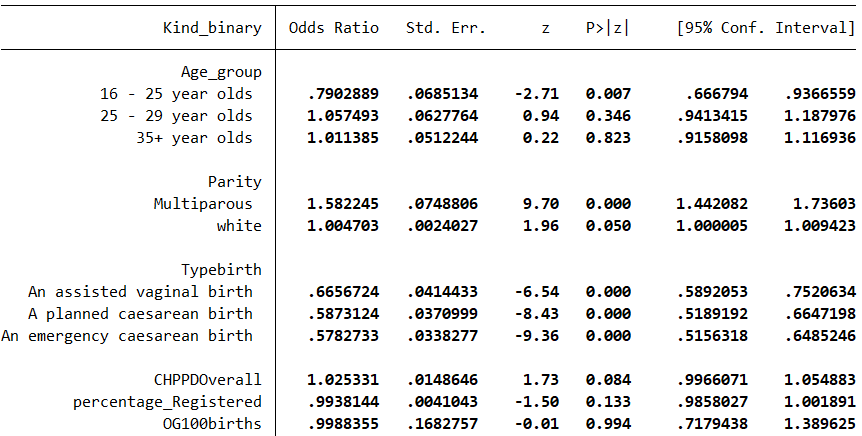


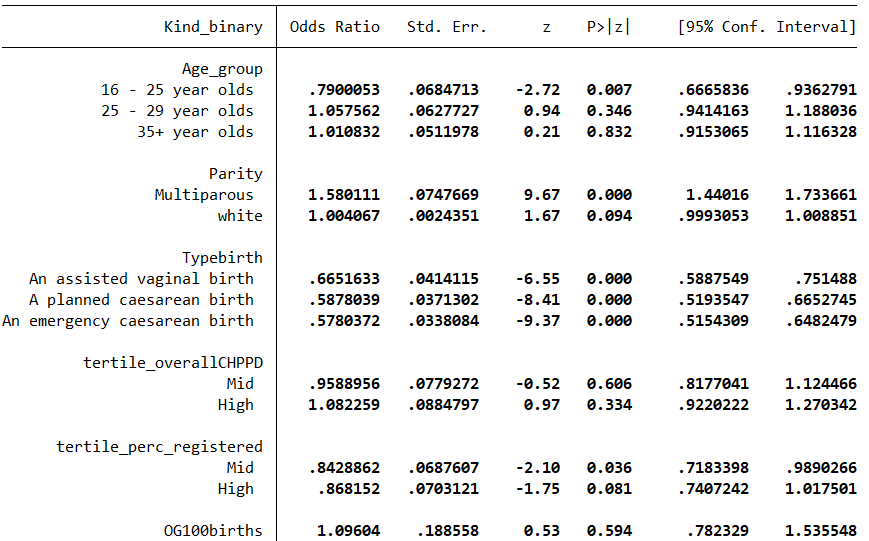

Supplement: S8 File — (DOCX) [file pone.0266638.s008.docx]
