# Supplementary material for: Exploring the relationship between women’s experience of postnatal care and reported staffing measures: An observational study
Source: PLoS One. 2022 Aug 2;17(8):e0266638. doi: 10.1371/journal.pone.0266638 (PMC9345482; doi:10.1371/journal.pone.0266638)
Supplement: S9 File — (DOCX) [file pone.0266638.s009.docx]

S9 Coding

use 2019 Edited_data_mat_survey_13,264x93 subset.dta

**NULL MODEL AND ESTIMATE CLUSTERING EFFECTS BY TRUST**

melogit No_delay_binary ||TrustCode:, or
estat ic
estat ic, n(93)
estat icc
melogit Help_binary ||TrustCode:, or
estat ic
estat ic, n(93)
estat icc
melogit Info_binary ||TrustCode:, or
estat ic
estat ic, n(93)
estat icc
melogit Kind_binary ||TrustCode:, or
estat ic
estat ic, n(93)
estat icc

**TRUST MEASURES OF STAFFING
With covariates explained in methodology**
melogit No_delay_binary ib3.Age_group i.Parity white i.Typebirth FTEper100births ||TrustCode:, or
estat ic
estat ic, n(93)
melogit Help_binary ib3.Age_group i.Parity white i.Typebirth FTEper100births ||TrustCode:, or
estat ic
estat ic, n(93)
melogit Info_binary ib3.Age_group i.Parity white i.Typebirth FTEper100births ||TrustCode:, or
estat ic
estat ic, n(93)
melogit Kind_binary ib3.Age_group i.Parity white i.Typebirth FTEper100births ||TrustCode:, or
estat ic
estat ic, n(93)

melogit No_delay_binary ib3.Age_group i.Parity white i.Typebirth i.tertile_FTEper100births ||TrustCode:, or
melogit Help_binary ib3.Age_group i.Parity white i.Typebirth i.tertile_FTEper100births ||TrustCode:, or
melogit Info_binary ib3.Age_group i.Parity white i.Typebirth i.tertile_FTEper100births ||TrustCode:, or
melogit Kind_binary ib3.Age_group i.Parity white i.Typebirth i.tertile_FTEper100births ||TrustCode:, or

**Testing model fit with additional covariates**melogit No_delay_binary ib3.Age_group i.Parity white i.Typebirth FTEper100births **Response_rate** ||TrustCode:, or
estat ic
estat ic, n(93)
melogit Help_binary ib3.Age_group i.Parity white i.Typebirth FTEper100births **Response_rate** ||TrustCode:, or
estat ic
estat ic, n(93)
melogit Info_binary ib3.Age_group i.Parity white i.Typebirth FTEper100births **Response_rate** ||TrustCode:, or
estat ic
estat ic, n(93)
melogit Kind_binary ib3.Age_group i.Parity white i.Typebirth FTEper100births **Response_rate**||TrustCode:, or
estat ic
estat ic, n(93)

melogit No_delay_binary ib3.Age_group i.Parity white i.Typebirth FTEper100births **annualbirthsHES**||TrustCode:, or
estat ic
estat ic, n(93)
melogit Help_binary ib3.Age_group i.Parity white i.Typebirth FTEper100births **annualbirthsHES** ||TrustCode:, or
estat ic
estat ic, n(93)
melogit Info_binary ib3.Age_group i.Parity white i.Typebirth FTEper100births **annualbirthsHES** ||TrustCode:, or
estat ic
estat ic, n(93)
melogit Kind_binary ib3.Age_group i.Parity white i.Typebirth FTEper100births **annualbirthsHES** ||TrustCode:, or
estat ic
estat ic, n(93)

melogit No_delay_binary ib3.Age_group i.Parity white i.Typebirth FTEper100births **OG100births** ||TrustCode:, or
estat ic
estat ic, n(93)
melogit Help_binary ib3.Age_group i.Parity white i.Typebirth FTEper100births **OG100births** ||TrustCode:, or
estat ic
estat ic, n(93)
melogit Info_binary ib3.Age_group i.Parity white i.Typebirth FTEper100births **OG100births** ||TrustCode:, or
estat ic
estat ic, n(93)
melogit Kind_binary ib3.Age_group i.Parity white i.Typebirth FTEper100births **OG100births** ||TrustCode:, or
estat ic
estat ic, n(93)

**POSTNATAL WARD STAFFING MEASURE**

**Staff as individual staff groups**

melogit No_delay_binary ib3.Age_group i.Parity white i.Typebirth CHPPDRegisteredNursesandMi CHPPDHealthcareSupportWorke ||TrustCode:, or
estat ic
estat ic, n(93)
melogit Help_binary ib3.Age_group i.Parity white i.Typebirth CHPPDRegisteredNursesandMi CHPPDHealthcareSupportWorke ||TrustCode:, or
estat ic
estat ic, n(93)
melogit Info_binary ib3.Age_group i.Parity white i.Typebirth CHPPDRegisteredNursesandMi CHPPDHealthcareSupportWorke ||TrustCode:, or
estat ic
estat ic, n(93)
melogit Kind_binary ib3.Age_group i.Parity white i.Typebirth CHPPDRegisteredNursesandMi CHPPDHealthcareSupportWorke ||TrustCode:, or
estat ic
estat ic, n(93)

**Testing model fit with additional covariates**melogit No_delay_binary ib3.Age_group i.Parity white i.Typebirth CHPPDRegisteredNursesandMi CHPPDHealthcareSupportWorke **Response_rate** ||TrustCode:, or
estat ic
estat ic, n(93)
melogit Help_binary ib3.Age_group i.Parity white i.Typebirth CHPPDRegisteredNursesandMi CHPPDHealthcareSupportWorke **Response_rate** ||TrustCode:, or
estat ic
estat ic, n(93)
melogit Info_binary ib3.Age_group i.Parity white i.Typebirth CHPPDRegisteredNursesandMi CHPPDHealthcareSupportWorke **Response_rate** ||TrustCode:, or
estat ic
estat ic, n(93)
melogit Kind_binary ib3.Age_group i.Parity white i.Typebirth CHPPDRegisteredNursesandMi CHPPDHealthcareSupportWorke **Response_rate** ||TrustCode:, or
estat ic
estat ic, n(93)

melogit No_delay_binary ib3.Age_group i.Parity white i.Typebirth CHPPDRegisteredNursesandMi CHPPDHealthcareSupportWorke **annualbirthsHES** ||TrustCode:, or
estat ic
estat ic, n(93)
melogit Help_binary ib3.Age_group i.Parity white i.Typebirth CHPPDRegisteredNursesandMi CHPPDHealthcareSupportWorke **annualbirthsHES** ||TrustCode:, or
estat ic
estat ic, n(93)
melogit Info_binary ib3.Age_group i.Parity white i.Typebirth CHPPDRegisteredNursesandMi CHPPDHealthcareSupportWorke **annualbirthsHES** ||TrustCode:, or
estat ic
estat ic, n(93)
melogit Kind_binary ib3.Age_group i.Parity white i.Typebirth CHPPDRegisteredNursesandMi CHPPDHealthcareSupportWorke **annualbirthsHES** ||TrustCode:, or
estat ic
estat ic, n(93)

melogit No_delay_binary ib3.Age_group i.Parity white i.Typebirth CHPPDRegisteredNursesandMi CHPPDHealthcareSupportWorke **OG100births** ||TrustCode:, or
estat ic
estat ic, n(93)
melogit Help_binary ib3.Age_group i.Parity white i.Typebirth CHPPDRegisteredNursesandMi CHPPDHealthcareSupportWorke **OG100births** ||TrustCode:, or
estat ic
estat ic, n(93)
melogit Info_binary ib3.Age_group i.Parity white i.Typebirth CHPPDRegisteredNursesandMi CHPPDHealthcareSupportWorke **OG100births** ||TrustCode:, or
estat ic
estat ic, n(93)
melogit Kind_binary ib3.Age_group i.Parity white i.Typebirth CHPPDRegisteredNursesandMi CHPPDHealthcareSupportWorke **OG100births** ||TrustCode:, or
estat ic
estat ic, n(93)
**This model has been reported in the paper as medical staff has improved model fit in ward level models.**

Overall staffing

**With covariates explained in methodology**

melogit No_delay_binary ib3.Age_group i.Parity white i.Typebirth CHPPDOverall percentage_Registered ||TrustCode:, or
estat ic
estat ic, n(93)
melogit Help_binary ib3.Age_group i.Parity white i.Typebirth CHPPDOverall percentage_Registered ||TrustCode:, or
estat ic
estat ic, n(93)
melogit Info_binary ib3.Age_group i.Parity white i.Typebirth CHPPDOverall percentage_Registered ||TrustCode:, or
estat ic
estat ic, n(93)
melogit Kind_binary ib3.Age_group i.Parity white i.Typebirth CHPPDOverall percentage_Registered ||TrustCode:, or
estat ic
estat ic, n(93)

**Testing model fit with additional covariates**
melogit No_delay_binary ib3.Age_group i.Parity white i.Typebirth CHPPDOverall percentage_Registered **Response_rate** ||TrustCode:, or
estat ic
estat ic, n(93)
melogit Help_binary ib3.Age_group i.Parity white i.Typebirth CHPPDOverall percentage_Registered **Response_rate** ||TrustCode:, or
estat ic
estat ic, n(93)
melogit Info_binary ib3.Age_group i.Parity white i.Typebirth CHPPDOverall percentage_Registered **Response_rate** ||TrustCode:, or
estat ic
estat ic, n(93)
melogit Kind_binary ib3.Age_group i.Parity white i.Typebirth CHPPDOverall percentage_Registered **Response_rate** ||TrustCode:, or
estat ic
estat ic, n(93)

melogit No_delay_binary ib3.Age_group i.Parity white i.Typebirth CHPPDOverall percentage_Registered **annualbirthsHES** ||TrustCode:, or
estat ic
estat ic, n(93)
melogit Help_binary ib3.Age_group i.Parity white i.Typebirth CHPPDOverall percentage_Registered **annualbirthsHES** ||TrustCode:, or
estat ic
estat ic, n(93)
melogit Info_binary ib3.Age_group i.Parity white i.Typebirth CHPPDOverall percentage_Registered **annualbirthsHES** ||TrustCode:, or
estat ic
estat ic, n(93)
melogit Kind_binary ib3.Age_group i.Parity white i.Typebirth CHPPDOverall percentage_Registered **annualbirthsHES** ||TrustCode:, or
estat ic
estat ic, n(93)

melogit No_delay_binary ib3.Age_group i.Parity white i.Typebirth CHPPDOverall percentage_Registered **OG100births** ||TrustCode:, or
estat ic
estat ic, n(93)
melogit Help_binary ib3.Age_group i.Parity white i.Typebirth CHPPDOverall percentage_Registered **OG100births** ||TrustCode:, or
estat ic
estat ic, n(93)
melogit Info_binary ib3.Age_group i.Parity white i.Typebirth CHPPDOverall percentage_Registered **OG100births** ||TrustCode:, or
estat ic
estat ic, n(93)
melogit Kind_binary ib3.Age_group i.Parity white i.Typebirth CHPPDOverall percentage_Registered **OG100births** ||TrustCode:, or
estat ic
estat ic, n(93)

Repeated analyses with staffing in tertiles

melogit No_delay_binary ib3.Age_group i.Parity white i.Typebirth i.tertile_FTEper100births ||TrustCode:, or
melogit Help_binary ib3.Age_group i.Parity white i.Typebirth i.tertile_FTEper100births ||TrustCode:, or
melogit Info_binary ib3.Age_group i.Parity white i.Typebirth i.tertile_FTEper100births ||TrustCode:, or
melogit Kind_binary ib3.Age_group i.Parity white i.Typebirth i.tertile_FTEper100births ||TrustCode:, or

melogit No_delay_binary ib3.Age_group i.Parity white i.Typebirth i.tertile_overallCHPPD i.tertile_perc_registered OG100births ||TrustCode:, or
melogit Help_binary ib3.Age_group i.Parity white i.Typebirth i.tertile_overallCHPPD i.tertile_perc_registered OG100births ||TrustCode:, or
melogit Info_binary ib3.Age_group i.Parity white i.Typebirth i.tertile_overallCHPPD i.tertile_perc_registered OG100births ||TrustCode:, or
melogit Kind_binary ib3.Age_group i.Parity white i.Typebirth i.tertile_overallCHPPD i.tertile_perc_registered OG100births ||TrustCode:, or

melogit No_delay_binary ib3.Age_group i.Parity white i.Typebirth i.TertileRegCHPPD i.TertileSupportCHPPD OG100births ||TrustCode:, or
melogit Help_binary ib3.Age_group i.Parity white i.Typebirth i.TertileRegCHPPD i.TertileSupportCHPPD OG100births ||TrustCode:, or
melogit Info_binary ib3.Age_group i.Parity white i.Typebirth i.TertileRegCHPPD i.TertileSupportCHPPD OG100births ||TrustCode:, or
melogit Kind_binary ib3.Age_group i.Parity white i.Typebirth i.TertileRegCHPPD i.TertileSupportCHPPD OG100births ||TrustCode:, or

**Sensitivity analyses for removing outliers**

melogit No_delay_binary ib3.Age_group i.Parity white i.Typebirth CHPPDRegOutliersremoved CHPPDSupportOutliersremoved OG100births ||TrustCode:, or

melogit Help_binary ib3.Age_group i.Parity white i.Typebirth CHPPDRegOutliersremoved CHPPDSupportOutliersremoved OG100births ||TrustCode:, or

melogit Info_binary ib3.Age_group i.Parity white i.Typebirth CHPPDRegOutliersremoved CHPPDSupportOutliersremoved OG100births ||TrustCode:, or

melogit Kind_binary ib3.Age_group i.Parity white i.Typebirth CHPPDRegOutliersremoved CHPPDSupportOutliersremoved OG100births ||TrustCode:, or

**Sensitivity analysis using alternative coding of yes and no for the models presented in the paper**

melogit Help_alternative ib3.Age_group i.Parity white i.Typebirth FTEper100births ||TrustCode:, or

melogit Info_alternative ib3.Age_group i.Parity white i.Typebirth FTEper100births ||TrustCode:, or

melogit Kind_alternative ib3.Age_group i.Parity white i.Typebirth FTEper100births ||TrustCode:, or

melogit Help_alternative ib3.Age_group i.Parity white i.Typebirth CHPPDRegisteredNursesandMi CHPPDHealthcareSupportWorke OG100births ||TrustCode:, or

melogit Info_alternative ib3.Age_group i.Parity white i.Typebirth CHPPDRegisteredNursesandMi CHPPDHealthcareSupportWorke OG100births ||TrustCode:, or

melogit Kind_alternative ib3.Age_group i.Parity white i.Typebirth CHPPDRegisteredNursesandMi CHPPDHealthcareSupportWorke OG100births ||TrustCode:, or

**Interactions**

melogit No_delay_binary ib3.Age_group i.Parity white i.Typebirth i.TertileRegCHPPD i.TertileSupportCHPPD OG100births ||TrustCode:, or
estat ic
estat ic, n(93)

melogit No_delay_binary ib3.Age_group i.Parity white i.Typebirth **i.TertileRegCHPPD##i.TertileSupportCHPPD** OG100births ||TrustCode:, or
estat ic
estat ic, n(93)

melogit Help_binary ib3.Age_group i.Parity white i.Typebirth i.TertileRegCHPPD i.TertileSupportCHPPD OG100births ||TrustCode:, or
estat ic
estat ic, n(93)

melogit Help_binary ib3.Age_group i.Parity white i.Typebirth **i.TertileRegCHPPD##i.TertileSupportCHPPD** OG100births ||TrustCode:, or
estat ic
estat ic, n(93)

melogit Info_binary ib3.Age_group i.Parity white i.Typebirth i.TertileRegCHPPD i.TertileSupportCHPPD OG100births ||TrustCode:, or
estat ic
estat ic, n(93)

melogit Info_binary ib3.Age_group i.Parity white i.Typebirth **i.TertileRegCHPPD##i.TertileSupportCHPPD** OG100births ||TrustCode:, or
estat ic
estat ic, n(93)

melogit Kind_binary ib3.Age_group i.Parity white i.Typebirth i.TertileRegCHPPD i.TertileSupportCHPPD OG100births ||TrustCode:, or
estat ic
estat ic, n(93)

melogit Kind_binary ib3.Age_group i.Parity white i.Typebirth **i.TertileRegCHPPD##i.TertileSupportCHPPD** OG100births ||TrustCode:, or
estat ic
estat ic, n(93)
