# Supplementary material for: Exploring the relationship between women’s experience of postnatal care and reported staffing measures: An observational study
Source: PLoS One. 2022 Aug 2;17(8):e0266638. doi: 10.1371/journal.pone.0266638 (PMC9345482; doi:10.1371/journal.pone.0266638)

## S10. Sensitivity analysis after removing outliers

Mild outliers for CHPPD were removed when they were more than 1.5 x IQR more unusual than Q1 or Q3. This resulted in the removal of data for 2 Trusts for Registered CHPPD data, no outlying Trusts for Support CHPPD data, and 1 Trust for Overall CHPPD data. There were no Extreme outliers classified as 3 x IQR more unusual than Q1 or Q3

Method of identifying outliers taken from Dunn P. Scientific Research and Methodology : An introduction to quantitative research and statistics in science, engineering and health 2021.<https://bookdown.org/pkaldunn/Book/identifying-outliers.html>

Removing the Trusts meant that individual patient data was removed from the analysis.
The models have been repeated using data with outlying values removed and treated as missing.

**Question related to being Discharged without delay**


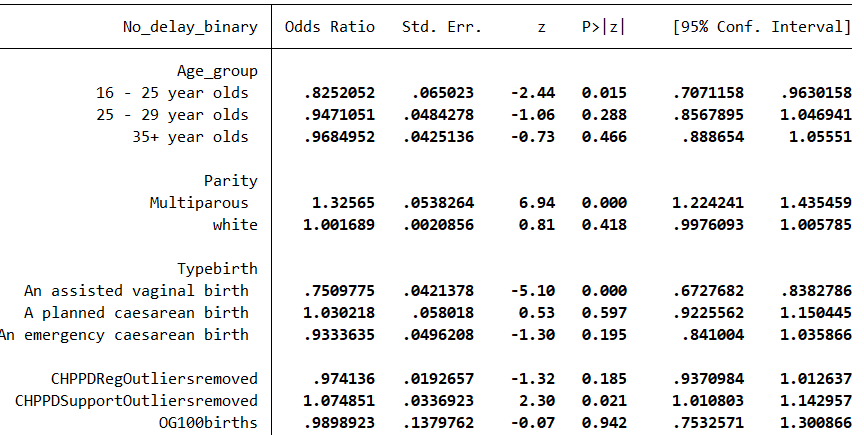


**Question related to Always having help when needed it**


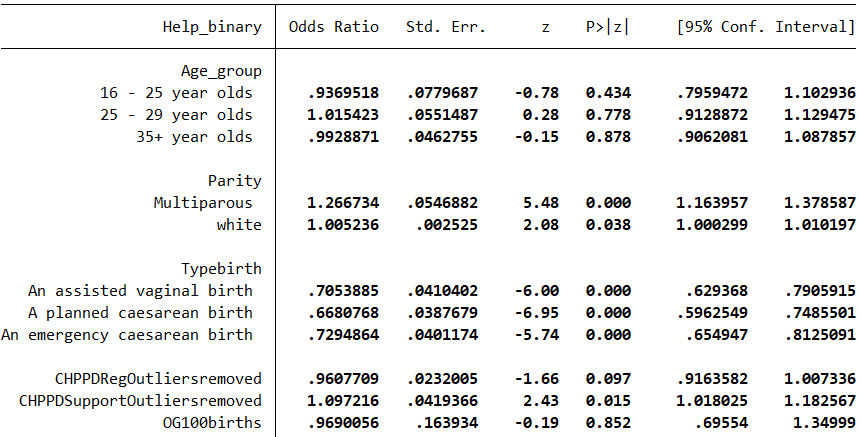


**Question related to Always having Info and explanations**


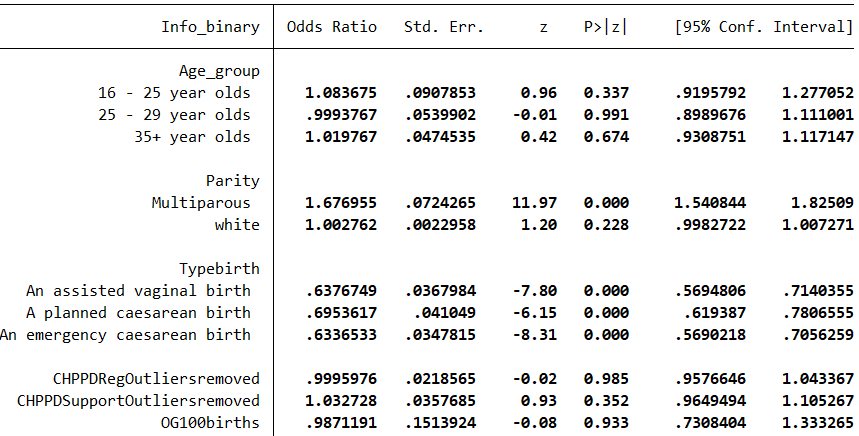


**Question related to Always being treated kindness and understanding**


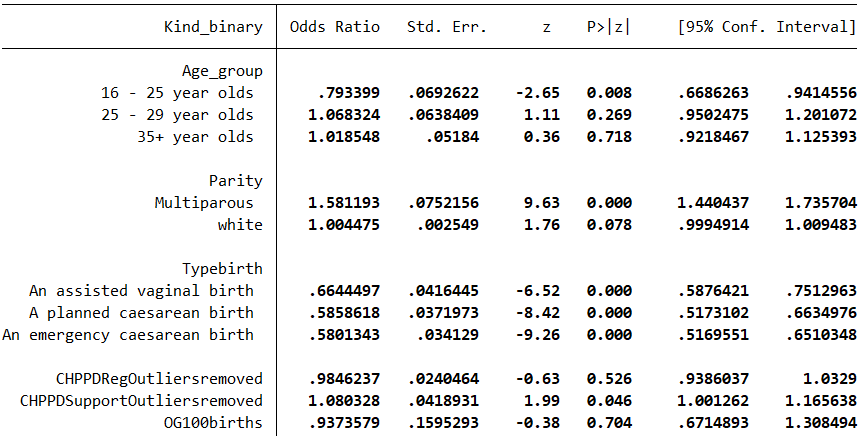

Supplement: S10 File — (DOCX) [file pone.0266638.s010.docx]
