# Supplementary material for: Exploring the relationship between women’s experience of postnatal care and reported staffing measures: An observational study
Source: PLoS One. 2022 Aug 2;17(8):e0266638. doi: 10.1371/journal.pone.0266638 (PMC9345482; doi:10.1371/journal.pone.0266638)
Supplement: S11 File — (DOCX) [file pone.0266638.s011.docx]

## S11. Sensitivity analysis : Testing model fit using AIC and BIC when including interaction variables (improvement if reduces AIC or BIC by >2)

|  | Continuous CHPPD models | | **Best fit?** |
| --- | --- | --- | --- |
| Delay in discharge | Core model with no interaction variables   \|  \|  \|  \| \| --- \| --- \| --- \| | AIC 16864.26  BIC 16975.61 BIC (93) 16902.25 | Reference |
|  | With interaction variable RegisteredxSupport Workers | AIC 16862.83 BIC 17003.87 BIC (93) 16910.95 | No improvement in model fit |
|  |  | |  |
| Help in reasonable time | Core model with no interaction variables | AIC 15209.34 BIC 15319.69 BIC (93) 15247.33 | Reference |
|  | With interaction variable RegisteredxSupport Workers | AIC 15212.78 BIC 15352.56 BIC (93) 15260.89 | No improvement in model fit |
|  |  | |  |
| Information/ Explanations | Core model with no interaction variables | AIC 15477.82 BIC 15589.1 BIC (93) 15515.81 | Reference |
|  | With interaction variable RegisteredxSupport Workers | AIC 15484  BIC 15624.96 BIC (93) 15532.12 | No improvement in model fit |
|  |  | |  |
| Treated with kindness and understanding | Core model with no interaction variables | AIC 13546.15  BIC 13657.5 BIC (93) 13584.14 | Reference |
|  | With interaction variable RegisteredxSupport Workers | AIC 13552.3 BIC 13693.35 BIC (93) 13600.42 | No improvement in model fit |
