# Supplementary material for: Exploring the relationship between women’s experience of postnatal care and reported staffing measures: An observational study
Source: PLoS One. 2022 Aug 2;17(8):e0266638. doi: 10.1371/journal.pone.0266638 (PMC9345482; doi:10.1371/journal.pone.0266638)

## S12. Sensitivity analyses using alternative dichotomy of question responses

The models have been repeated using data with recoding of responses,
with yes always / yes sometimes = 1, no=0

**Help when you needed it?**


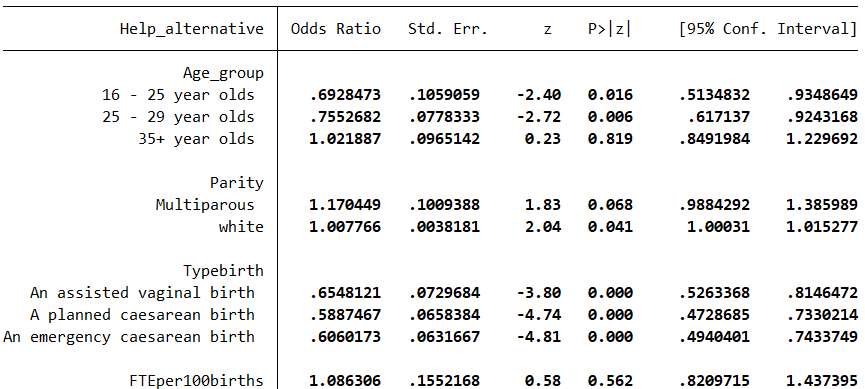


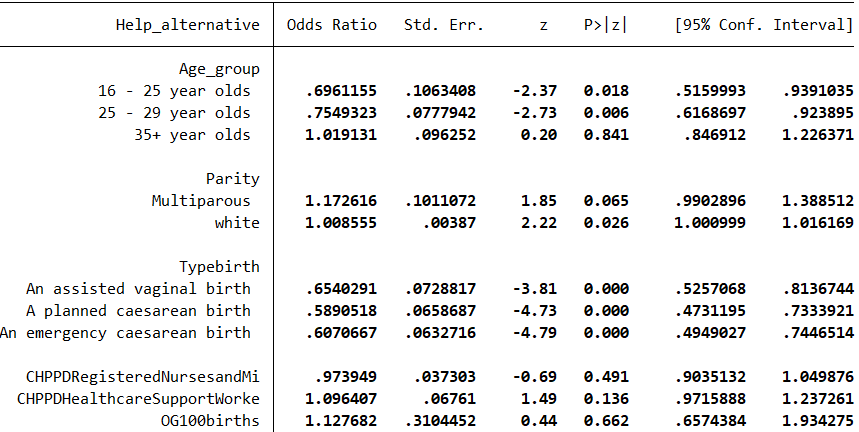


**Given the information or explanations you needed?**


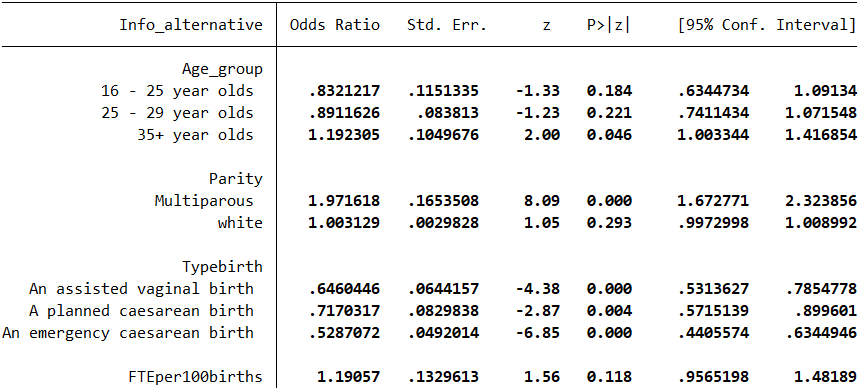


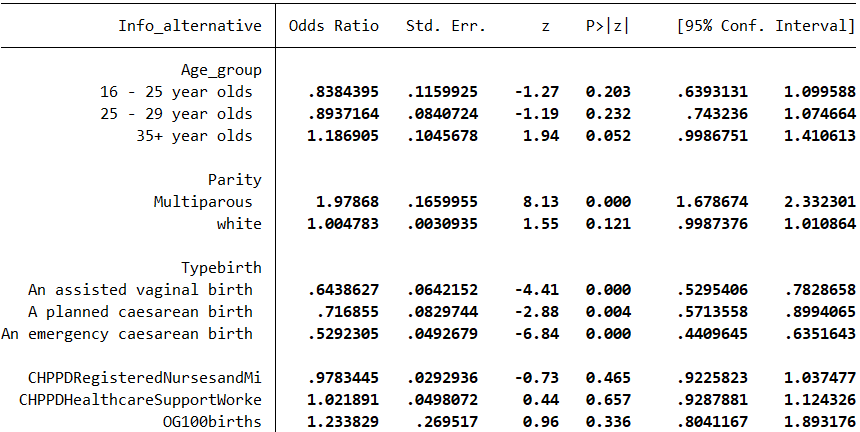


**Treated with kindness and understanding?**


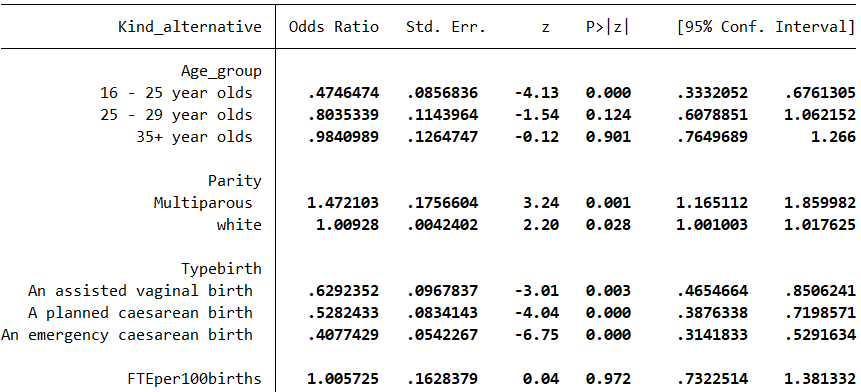


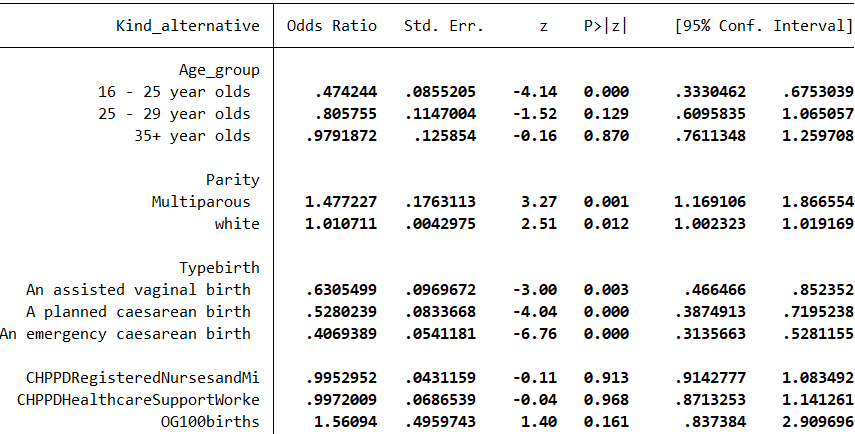

Supplement: S12 File — (DOCX) [file pone.0266638.s012.docx]
