# Supplementary material for: Exploring the relationship between women’s experience of postnatal care and reported staffing measures: An observational study
Source: PLoS One. 2022 Aug 2;17(8):e0266638. doi: 10.1371/journal.pone.0266638 (PMC9345482; doi:10.1371/journal.pone.0266638)
Supplement: S13 File — (DOCX) [file pone.0266638.s013.docx]

## S13 Null models for four questions and intra-class correlation coefficients

**Question related to being Discharged without delay**
melogit No_delay_binary ||TrustCode:, or


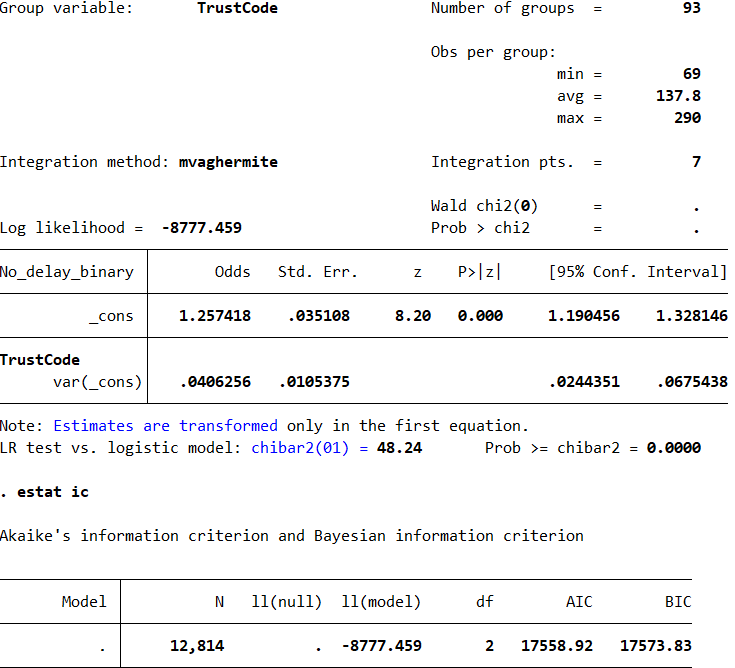


**Question related to Always having help when needed it**melogit Help_binary ||TrustCode:, or


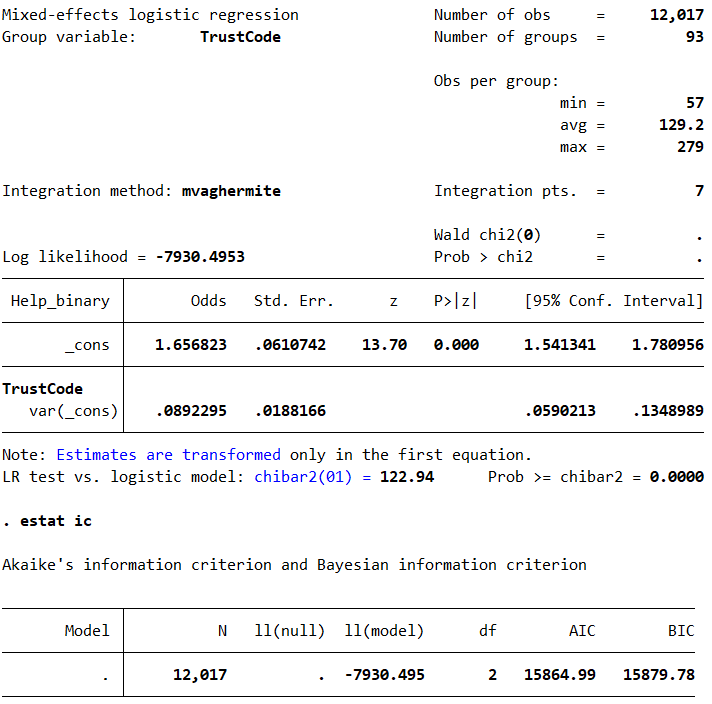


**Question related to Always having Info and explanations**

melogit Info_binary ||TrustCode:, or


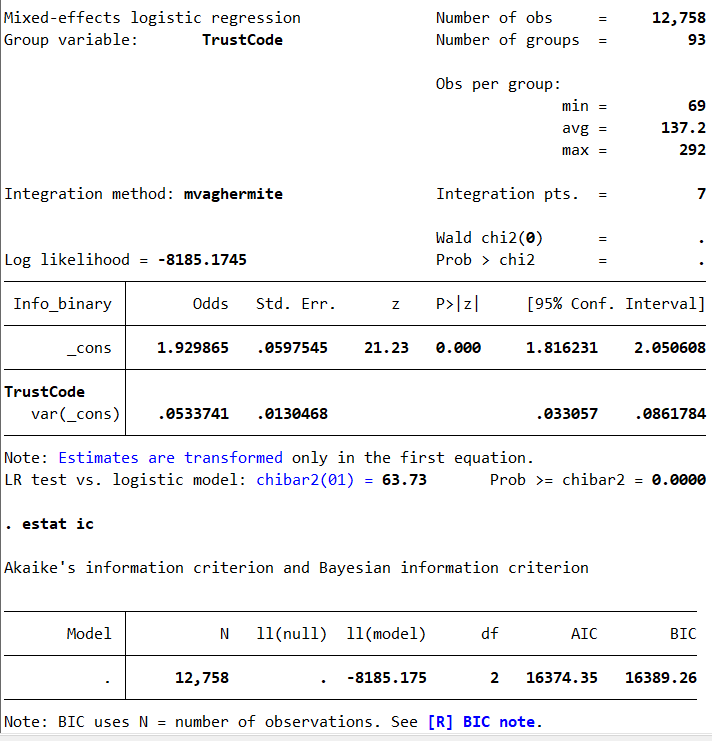


**Question related to Always being treated kindness and understanding**melogit Kind_binary ||TrustCode:, or


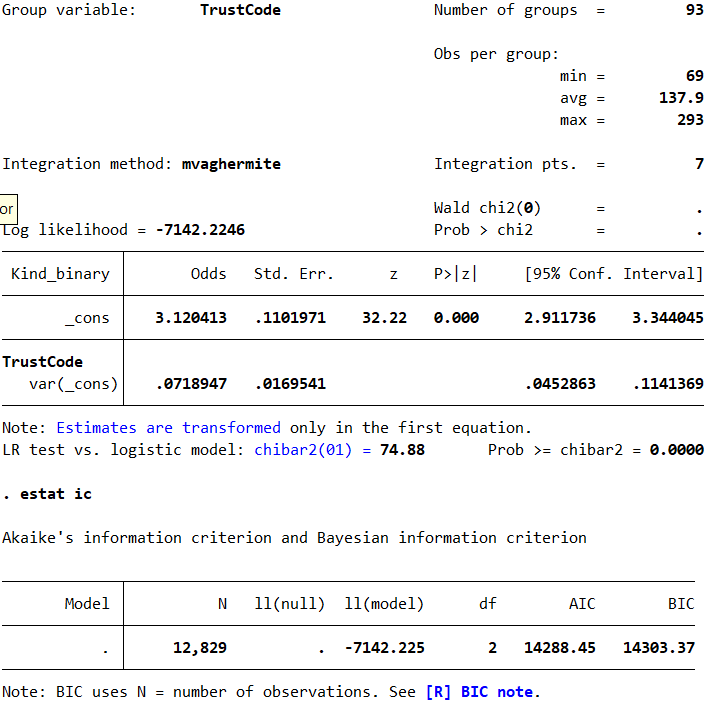


The **Intra-class Correlation Coefficient (ICC)** represents the dependence of scores between individuals in the same group, in this case meaning the level 2 variables (Trusts). If the coefficient value is high (>.10), a multilevel analysis is extremely important to reduce the risk of type I error. For each of the four outcomes the ICC is less than 0.03 which shows a relatively low level of inter-dependence of observations within groups (lack of extreme clustering). We can conclude that most of variation is within the clusters rather than between clusters. Non-hierarchical models could have been used with similar results, as the clustering effect is low in this data.

|  | Delay in discharge | Staff help reasonable time | Information / Explanations | Treated with kindness and understanding |
| --- | --- | --- | --- | --- |
| ICC | 0.0122 | 0.0264 | 0.0160 | 0.0214 |
