# Supplementary material for: Exploring the relationship between women’s experience of postnatal care and reported staffing measures: An observational study
Source: PLoS One. 2022 Aug 2;17(8):e0266638. doi: 10.1371/journal.pone.0266638 (PMC9345482; doi:10.1371/journal.pone.0266638)
Supplement: Attachment — Submitted filename: Review PLOS ONE.pdf [file pone.0266638.s014.pdf]

Review on manuscript "PONE-D-22-08119: Exploring the relationship between women's experience of postnatal care and reported staffing measures: an observational study"

The study deals with a highly interesting topic which fits well in the literature stream on hospital staffing and quality of care. With the focus on midwife staffing and quality of postnatal care, it tackles an area which has not been as well researched as others and yet is not less relevant. I have several comments on how to improve the paper, which I will outline in the order of the paper.

Introduction/Research question

In the introduction, the authors elaborate on the relevance of the research, present the state of the art and the objectives of the study. In my view, the structure could still be improved; it feels like the paper is jumping back and forth between practical relevance and prior evidence. Additionally, the authors could outline more explicitly the research gap and the contribution of the study.

The research questions bring up an issue that "haunted" me throughout the paper. In my view, the differentiation between organizational/trust level and ward level is misleading; it suggests that there is a different number of observations behind each level. However, as data on multiple postnatal wards is aggregated (i.e., averaged) on organizational level, the n should be identical. Thus, it is not really – or at least, not only – a question on the level of the data, but on the specific staffing measure and the affiliation/assignment to the postnatal care department(s).

In that sense, the measure midwives per 100 births is less precise because it is 1. a yearly average, 2. it does not account for variations in patient days, and 3. because it is not able to measure how many midwives were deployed in the postnatal ward area. I urge the authors to delineate more in-depth in how far the measures differ, how this affects interpretation, and whether it is really a question of aggregation/disaggregation, which the authors refer to several times (the study by Winter et. al. 2021 [23] demonstrate what happens (1) when aggregating data over the course of the month versus a year, (2) when estimating this relationship aggregated for the entire hospital, versus at the unit-level, with separate estimates for acute care units and ICUs, and (3) when using fixed effects to attempt to control for unobserved heterogeneity between units over time, versus no fixed effects. In my view, this is dissimilar to the data available here, which does not represent aggregation).

Methods/Results

Concerning the methods section, it seems that most relevant information is included. I would recommend a clearer structure again, potentially including subheadings for data sources, measures, sample description, and data analysis strategy. Again, the differentiation between ward and trust level remains confusing to me.

I further have some comments and suggestions for the data analyses:

1. I am somewhat in doubt that the authors change the covariates across the analyses; why not including them consistently across the different regressions?
2. Are the results of the null model reported? There should at least be a statement on the extent of between-trust variation in the outcomes.
3. I do not think that including the two staff groups as independent groups is purposeful. Due to the supplementary nature, I would assume that both coefficients should be expected to be positive (a higher number of registered staff (support staff) hours per patient day increases the quality of care when all other variables – including the number support staff (registered staff) hours per patient day – are held constant). In addition, dividing the sample into tertiles seems to mask information rather than ease interpretation. Thus, I would suggest reporting S9b (including covariates) as main analysis and results.

Discussion

The discussion needs to be revised considering the comments above, in particular, changes in the main data analysis and the interpretational differences between the staffing measures.

In addition, referring to “our study in the preceding year” and the following statements make it unclear what belongs to this study and what belongs to the older study. A clearer differentiation and comparison (previous findings on midwife staffing, this study’s findings on midwife staffing, and this study’s findings on ward CHPPD) should be made, also to underpin the value added by this study. Furthermore, the study’s finding should be framed more extensively in context of other prior evidence.

One major limitation of the study is its cross-sectional nature and the potential endogeneity issues, e.g., omitted variable bias or selection effects. These should be elaborated on.
