# Supplementary material for: Exploring the relationship between women’s experience of postnatal care and reported staffing measures: An observational study
Source: PLoS One. 2022 Aug 2;17(8):e0266638. doi: 10.1371/journal.pone.0266638 (PMC9345482; doi:10.1371/journal.pone.0266638)
Supplement: Attachment — Submitted filename: PlosOne 052022.docx [file pone.0266638.s015.docx]

Thank you for the opportunity to review this timely and interesting study. Overall, I think this is a useful addition to the literature, but I think there are ways the study could be improved. The most important of these would be to situate this paper more fully within the larger body of maternity staffing literature.

Introduction

- The problem you are describing is familiar in the US and it would be helpful to have some of the context explained to support a wider audience’s understanding. For example, a sentence describing Trusts, the relationship of trusts to wards (94 trusts with 124 postnatal wards?), the relationship of midwifery to nursing (roles and responsibilities), who is included in “support worker staffing” (are these nursing assistants, OR techs, etc.).

- p3 ln 48 – such work has also been done in the US (Kathleen Simpson and Audrey Lyndon)

- p 4 ln 59 – it might be more accurate to say that there is a confluence of system and individual level factors contributing to increased acuity (in the US, for example, racism contributes significantly to these outcomes, and trumps individual level factors). Chronological age in and of itself is not a risk factor; biological aging, to the extent that it leads to an increased incidence of obesity, diabetes, etc., might be.

- Consider briefly describing the difference between CHPPD and NHPPD to support reader understanding

- There’s a body of literature delineating the difficulties of measuring maternity staffing. Nursing hours per patient day hasn’t worked in a maternity setting previously – why use it here, consider justifying it, would it be possible to do sensitivity analyses with other measures?

Methods

- Is “age band” the same as “age category”?

- What are “bank” staff? Are these per diem/floaters? What is meant by supernumerary staff and how might their exclusion impact findings?

- Consider commenting in the discussion regarding not counting newborns and the impact of this on findings. If dyad care is the norm in the UK, not counting newborns might have a significant effect on the study’s findings.

- It looks as though there’s no way to separate out nursing from midwifery – is that correct? Again, considering commenting in discussion section re impact on outcomes

- p 9, ln 162-164 – In US-based research, the hospital where you give birth explains a significant proportion of the birth outcome (more so than individual-level characteristics) – is this not true in the UK? Again, racial disparities drive clinical outcomes and patience experience in the US and there’s evidence for this in the UK as well, (Knight 2020; Nellums 2021; Chitongo 2022), but it seems to be included here only as it improves fit. Might consider further discussion of this in discussion.

Results

- an aside: I was really surprised by the frequency of instrumental births. I had been under the impression that the UK had higher SVB rates than the US due, in part, to the more extensive presence of midwives. I’m intrigued to find that this is not the case in this sample.

- was ethnicity associated in any way to the postnatal experience measures?

- minor detail, but after reviewing the supplementary material, you might consider making age group of 30-34 the base category (since it’s the most common), at least, reconsider having the 16-25 year olds as the base category since they are the smallest group and teens tend to have their own issues re birth

Discussion

- Again, as in introduction, might consider broadening the discussion for a wider audience

- the lack of a strong relationship between trust and ward-level staffing is interesting and I’m glad to see it’s discussed. Consider discussing it further, however, and considering why this might be the case (especially as it was not what you expected). There’s far more staffing research that has been done in the US – and there’s work that is maternity specific that would be good to incorporate.

- There are several papers that have detailed the difficulties of measuring maternity staffing – and different attempts to measure it have been made – it would be good to incorporate and discuss those papers in light of your work.

- Consider discussing what impact the inability to disaggregate nurses and midwives at the ward-level might have had on outcomes. Also, the exclusion of newborns.

- there’s a structural/transactional piece that staffing seems to help with (discharge, information) that isn’t necessarily associated with improved interpersonal interactions (help when needed it, kindness) 🡪 interesting and might be worth further discussion; is this a function of the work environment (which has been shown to be more foundational than staffing in other research)?
