# Supplementary material for: Exploring the relationship between women’s experience of postnatal care and reported staffing measures: An observational study
Source: PLoS One. 2022 Aug 2;17(8):e0266638. doi: 10.1371/journal.pone.0266638 (PMC9345482; doi:10.1371/journal.pone.0266638)
Supplement: Attachment — Submitted filename: Response to reviewers.docx [file pone.0266638.s016.docx]

*Please note that line numbers refer to the Tracked changes copy (not the clean copy)*

**RESPONSE TO REVIEWER #1**

| The study deals with a highly interesting topic which fits well in the literature stream on hospital staffing and quality of care. With the focus on midwife staffing and quality of postnatal care, it tackles an area which has not been as well researched as others and yet is not less relevant. | Thank you for your time in reviewing our paper and for your helpful suggestions to improve it. We are pleased that you have endorsed that this is an important topic to study and meets a gap in the current research. |
| --- | --- |
| **Introduction/Research question** Structure could still be improved; it feels like the paper is jumping back and forth between practical relevance and prior evidence. Authors could outline more explicitly the research gap and the contribution of the study. | More evidence has been carefully added with structure in mind and carefully proof-read to improve flow  Paragraph added before the research question to highlight the contribution of this study - Line 108-112 |
| The differentiation between organizational/trust level and ward level is misleading; it suggests that there is a different number of observations behind each level. However, as data on multiple postnatal wards is aggregated (i.e., averaged) on organizational level, the n should be identical It is not really – or at least, not only – a question on the level of the data, but on the specific staffing measure and the affiliation/assignment to the postnatal care department | Thank you for pointing this out. Yes the *n* is identical as the organisational analysis has been conducted in the same Trusts as the measurement on the wards. The language used throughout the report has been adjusted to report the different staffing measures rather than referring to levels themselves. |
| I urge the authors to delineate more in‐depth in how far the measures differ, how this affects interpretation, and whether it is really a question of aggregation/disaggregation.    The study by Winter et. al. 2021 is dissimilar to the data available here. | Thank you for this point, we have added additional information into Line 136 onwards to explain how HPPD measures workload more precisely than organisational level staffing. We have removed reference to level and aggregation and instead talked of staffing measures (as in the response above)  We have removed the reference to Winter (lines 91-93 and 334-336) which discussed aggregation of staffing measures. This has been replaced with the term ‘precise’ as we agree that this is correct in terms of describing staffing levels closer to the patient rather than aggregation, as the number of data points are the same between Ward and Trust analyses. |
| **Methods/Results** It seems that most relevant information is included.  I would recommend a clearer structure again, potentially including subheadings for data sources, measures, sample description, and data analysis strategy. | Thank you for this suggestion, we have added sub headings into the methods to clarify the structure for readers. |
| **Data analyses** The authors change the covariates across the analyses; why not including them consistently across the different regressions? | Thank you for this advice. We have now included covariates consistently across the models and justified those included in the main analysis. This has led to slight alterations in the digits in Tables 5 and 6. Ethnicity has now been included in all models, and medical staff in all the models of ward staffing due to model fit. |
| Are the results of the null model reported? There should at least be a statement on the extent of between‐trust variation in the outcomes. | Thank you for this suggestion. We have added a supplementary file S13 with results of the null models with no covariates. The intra class correlation coefficients are low, which indicates that variability is likely to be within Trusts rather than between different Trusts (lack of strong clustering effects). |
| I do not think that including the two staff groups as independent groups is purposeful. Due to the supplementary nature, I would assume that both coefficients should be expected to be positive (a higher number of registered staff (support staff) hours per patient day increases the quality of care when all other variables – including the number support staff (registered staff) hours per patient day – are held constant).  Dividing the sample into tertiles seems to mask information rather than ease interpretation. I would suggest reporting S9b (including covariates) as main analysis and results. | Thank you for your comment. We had chosen to look at independent staffing groups first as the advantage of the HPPD data is that the support worker staffing can be studied and this is a new area. The potential for supplementary effects has been modelled using interaction terms in Supplementary material S11. These models did not improve model fit and therefore were not explored in greater depth. The result show that for registered staff the coefficients were not positive as expected and this is useful to describe, and this may have been hidden in Overall staffing alone.  Staffing as a continuous variable has now been added in the Tables 5 and 6 in the manuscript. We have retained the tertile analysis in the paper as some results comparing the highest and lowest staffed tertiles are remarked upon in the text. |
| **Discussion** The discussion needs to be revised considering the comments above, in particular, changes in the main data analysis and the interpretational differences between the staffing measures. | Thank you, the discussion has been revised with reference to the different staffing measures used in this study. |
| Referring to “our study in the preceding year” make it unclear what belongs to this study and what belongs to the older study. A clearer differentiation and comparison (previous findings on midwife staffing, this study’s findings on midwife staffing, and this study’s findings on ward CHPPD) should be made, also to underpin the value added by this study. | Thank you for this observation. The language has been tightened up in lines 340-344 to make this clearer. |
| The study’s finding should be framed more extensively in context of other prior evidence. | Thank you, new references have been added e.g. Simpson (2016), Simpson (2019), Birthrights (2022), MBRRACE (2021), Russell (2021), Twigg (2009), Henderson (2013), NMPA (2021). Some of these areas for discussion have been suggested by reviewer #2 as well as yourself. |
| One major limitation of the study is its cross‐sectional nature and the potential endogeneity issues, e.g., omitted variable bias or selection effects. These should be elaborated on. | We agree that these are important to include and have added text to lines 393-399 to elaborate on this. |

**RESPONSE TO REVIEWER #2**

| Thank you for the opportunity to review this timely and interesting study. Overall, I think this is a useful addition to the literature, but I think there are ways the study could be improved. The most important of these would be to situate this paper more fully within the larger body of maternity staffing literature. | Thank you for your time in reviewing our paper and for your helpful suggestions to improve it.   The reference list has been expanded to 58 references from 41, and we have included many more international references, particularly those on maternity staffing. |
| --- | --- |
| **Introduction** The problem you are describing is familiar in the US and it would be helpful to have some of the context explained to support a wider audience’s understanding. For example, a sentence describing Trusts, the relationship of trusts to wards (94 trusts with 124 postnatal wards?), the relationship of midwifery to nursing (roles and responsibilities), who is included in “support worker staffing” (are these nursing assistants, OR techs, etc.). | Thank you for highlighting this, we have added the following explanations:  Explanation of ward relationship to Trusts line 155-156  Midwifery and nursing roles added to lines 423-427  Role of Support workers outlined in line 96-98 |
| p3 ln 48 – such work has also been done in the US (Kathleen Simpson and Audrey Lyndon) | Thank you, this work has now been included and new references added from these authors (references 13 and 14) |
| p 4 ln 59 – it might be more accurate to say that there is a confluence of system and individual level factors contributing to increased acuity (in the US, for example, racism contributes significantly to these outcomes, and trumps individual level factors). Chronological age in and of itself is not a risk factor; biological aging, to the extent that it leads to an increased incidence of obesity, diabetes, etc., might be. | Thank you, we have amended this section to recognise a fuller range of factors influencing risk, including racial differences (lines 68-73) |
| Consider briefly describing the difference between CHPPD and NHPPD to support reader understanding | Features of HPPD listed in line 136-139 as it demarcates registered from support staff, which is something not always done in some NHPPD calculations. We have dropped the term CHPPD from the paper and only referred to it when describing the dataset as this is its name. We have instead referred to HPPD and highlighted whether this relates to registered staff or support staff. This is to bring the paper in line with the international literature. |
| There’s a body of literature delineating the difficulties of measuring maternity staffing. Nursing hours per patient day hasn’t worked in a maternity setting previously – why use it here, consider justifying it, would it be possible to do sensitivity analyses with other measures? | We have acknowledged that the maternity setting is different to medical and surgical wards where NHPPD is it usually used – line 105-106 We have also included the paper from Clark which outlines some of the problems in measuring maternity staffing – lines 418-421 |
| **Methods** Is “age band” the same as “age category”? | Thank you, yes these are the same. We have amended the words ‘band’ and ‘category’ to ‘group’ to align with the data in the supplementary material |
| What are “bank” staff? Are these per diem/floaters? What is meant by supernumerary staff and how might their exclusion impact findings? | This has been added to the discussion section (lines 394-399). It is not clear how the exclusion of additional staff would affect the findings but is a useful point to raise for the readers. |
| Consider commenting in the discussion regarding not counting newborns and the impact of this on findings. If dyad care is the norm in the UK, not counting newborns might have a significant effect on the study’s findings. | Thank you, we have now expanded on this point in lines 401-408 |
| It looks as though there’s no way to separate out nursing from midwifery – is that correct? Again, considering commenting in discussion section re impact on outcomes | This has been added to the discussion section as it is an emerging trend to employ more nurses in maternity services, and we were unable to separate these out. We have highlighted the importance of doing this in future studies (lines 427-430) |
| p 9, ln 162-164 – In US-based research, the hospital where you give birth explains a significant proportion of the birth outcome (more so than individual-level characteristics) – is this not true in the UK?  Again, racial disparities drive clinical outcomes and patience experience in the US and there’s evidence for this in the UK as well, (Knight 2020; Nellums 2021; Chitongo 2022), but it seems to be included here only as it improves fit. Might consider further discussion of this in discussion. | NHS hospitals work to similar protocols and standards of care (as many adopt NICE guidelines and are funded centrally). It is our understanding that most differences in outcomes are attributable to patient characteristics rather than between hospitals. In the Sandall (2014) study variation between Trusts represents a comparatively small component of the overall variation (approximately 1–2%) in outcomes.  Thank you, this has been added into the discussion and highlighted that ethnicity is an important variable to include given the differences in clinical outcomes and experiences documented in UK and internationally (lines 432-438). |
| **Results** an aside: I was really surprised by the frequency of instrumental births. I had been under the impression that the UK had higher SVB rates than the US due, in part, to the more extensive presence of midwives. I’m intrigued to find that this is not the case in this sample. | The respondents in our survey had slightly more instrumental births than our background population (14.7% instrumental birth in the survey population compared to 11% in February 2022 according to the Maternity services dataset). Other modes of birth were broadly similar to the population (55% spontaneous birth vs 53% in population), caesarean (30% vs 34% in population). We have not commented on this in the paper. |
| was ethnicity associated in any way to the postnatal experience measures? | Thank you for your query. This was tested in the univariable analysis and evidence of an effect was seen, although it was very small. However, we only had Trust level proportions for each ethnic group available which limits the ability to test this covariate. We have now included this as a covariate in all models given the strength of the literature and UV results. Thank you for highlighting this. |
| minor detail, but after reviewing the supplementary material, you might consider making age group of 30-34 the base category (since it’s the most common), at least, reconsider having the 16-25 year olds as the base category since they are the smallest group and teens tend to have their own issues re birth | Thank you for this suggestion, all supplementary material have been updated with the 30-34 age group as the base category. |
| **Discussion** Again, as in introduction, might consider broadening the discussion for a wider audience | Thank you for this suggestion. We have now discussed staff groups other than midwives, as midwives are not always employed internationally. We have also considered the exclusion of newborns and referred to couplet care in the USA. We have included more discussion about ethnicity and the relationship to outcomes and areas of incomplete care found in research studies. |
| The lack of a strong relationship between trust and ward-level staffing is interesting and I’m glad to see it’s discussed. Consider discussing it further, however, and considering why this might be the case (especially as it was not what you expected). There’s far more staffing research that has been done in the US – and there’s work that is maternity specific that would be good to incorporate. | Thank you, we have elaborated on this point in lines 332-334  We agree that more staffing research is needed in the discussion. We have included more research papers in the introduction and discussion, especially papers from the US where much of the research has been conducted. |
| There are several papers that have detailed the difficulties of measuring maternity staffing – and different attempts to measure it have been made – it would be good to incorporate and discuss those papers in light of your work. | Thank you for this suggestion, this point has now been included in lines 417-421. |
| Consider discussing what impact the inability to disaggregate nurses and midwives at the ward-level might have had on outcomes. Also, the exclusion of newborns. | We have expanded on this in lines 423-430 to explain that most of the registered staff in our study are likely to be midwives. We acknowledge that this pattern is changing and is not always the case in other parts of the world. |
| There’s a structural/transactional piece that staffing seems to help with (discharge, information) that isn’t necessarily associated with improved interpersonal interactions (help when needed it, kindness) 🡪 interesting and might be worth further discussion; is this a function of the work environment (which has been shown to be more foundational than staffing in other research)? | Thank you for this suggestion, unfortunately the word count has not enabled us to expand on this in detail. We have tried to cover this in terms of the support worker role contributing to improved responses on women receiving help when they needed it and being treated with kindness and understanding (lines 361-366). Your suggestion is something we could explore in future studies, thank you. |
